# Angioimmunoblastic T-cell lymphoma with predominant CD8+ tumor-infiltrating T-cells is a distinct immune pattern with an immunosuppressive microenvironment

**DOI:** 10.3389/fimmu.2022.987227

**Published:** 2022-10-17

**Authors:** Zihang Chen, Qiqi Zhu, Xueqin Deng, Wenqing Yao, Wenyan Zhang, Weiping Liu, Yuan Tang, Sha Zhao

**Affiliations:** Department of Pathology, West China Hospital, Sichuan University, Chengdu, China

**Keywords:** angioimmunoblastic T-cell lymphoma, tumor microenvironment, T cell receptor-β repertoire, immunoglobulin heavy chain repertoire, TME function

## Abstract

**Background:**

Angioimmunoblastic T-cell lymphoma (AITL) has a rich tumor microenvironment (TME) that typically harbors plenty of CD4+tumor infiltrating lymphocytes, (TIL)-T-cells (so called common AITL). Nonetheless, AITL with large numbers of CD8+TIL-Ts that outnumber CD4+cells have been observed (CD8-predominant AITL). However, detailed comparison of CD8-predominant AITL and common AITL are still lacking.

**Methods:**

We compared clinicopathological features, TIL subsets, TME T cell receptor-β (TRB), and immunoglobulin heavy chain (IGH) repertoires, and gene expression profiles in six CD8-predominant and 12 common AITLs using case-control matching (2014 to 2019).

**Results:**

Comparing with common AITLs, CD8-predominant AITLs showed more frequent edema (*P =* 0.011), effusion (*P =* 0.026), high elevated plasma EBV-DNA (P = 0.008), and shorter survival (P = 0.034). Moreover, they had more pronounced eosinophil increase (P = 0.004) and a higher Ki67 index (P = 0.041). Flow cytometry revealed an inverted CD4/CD8 ratio in TIL-Ts and lower TIL-B proportions (P = 0.041). TRB repertoire metrics deteriorated, including lower productive clones (P = 0.014) and higher clonality score (P = 0.019). The IGH repertoire was also narrowed, showing a higher proportion of the top 10 clones (P = 0.002) and lower entropy (P = 0.027). Gene expression analysis showed significant enrichment for upregulated negative regulation of immune system processes and downregulated T-cell activation and immune cell differentiation.

**Conclusion:**

Our findings demonstrated that CD8-predominant AITL is a distinct immune pattern of AITL characterized by anti-tumor immunity impairment and an immunosuppressive microenvironment. These characteristics can interpret its severe clinical manifestations and poor outcomes.

## Introduction

Angioimmunoblastic T-cell lymphoma (AITL) is a subtype of peripheral T-cell lymphoma (PTCL) with a T follicular helper (TFH) phenotype (1). In our previous analysis, it was the fourth most common mature T-cell lymphoma, accounting for 11% of all patients with PTCL (2). Patients with AITL frequently exhibit clinical symptoms, including skin rash, arthritis, effusion, and positive autoimmune tests, indicating an aberrant immune response (3, 4). Finally, patients exhibit immunodeficiency secondary to neoplastic development. Even following an intensive treatment regimen, the prognosis is generally poor, with most studies reporting a median survival of < 3 years (5). As a result, immunological dysregulation characterizes the entire course of AITL and has been identified as a distinguishing feature of this malignancy.

AITL possesses a complex tumor microenvironment (TME) composed of irregularly proliferating follicular dendritic cells in the meshwork, arborizing high endothelial venules (HEVs) and a variety of reactive immune cells, the most obvious of which are B-immunoblasts, plasma cells, and tumor-infiltrating lymphocyte (TIL) -T cells (TIL-Ts) (6). Immunostaining has revealed that AITL TIL-Ts often have a significantly larger proportion of CD4+ cells than CD8+ cells (defined as common AITL in our study), which is due to CD4 expression in neoplastic T-cells and a normal CD4+/CD8+ ratio in TIL-Ts (CD4+TIL-Ts outnumber CD8+TIL-Ts) (7, 8). Nonetheless, we observed a few cases of AITL with an extremely inverted CD4/CD8 ratio (defined as CD8-predominant AITL in our study), indicating the significant increase of CD8+TIL-Ts. However, CD8-predominant AITL was rare, accounting for only 1.5% of AITL cases in daily practice (Time: 2014-2019).

The different CD8+ TIL-T proportions in the TMEs of CD8-predominant AITL and common AITL implied that TME immune function differed between the two AITL groups (7). Furthermore, the immune function of the TME not only correlated with the patient’s clinical manifestations related to inflammation and immune response but also reflects anti-tumor immunity, which has a direct impact on tumor development and progression. However, detailed comparison of CD8-predominant AITL and common AITL are still lacking. Therefore, we used a case-control matching approach to include cohorts of CD8-predominant AITL and common AITL cases for clinicopathological analysis, flow cytometry testing, T cell receptor-β (TRB), immunoglobulin heavy chain (IGH) repertoire sequencing, and RNA sequencing. Data were compared to gain a better understanding of the TME immune function of this uncommon CD8-predominant AITL and its connection with clinicopathological findings.

## Methods

### Case selection

The CD8-predominant AITL cohort was identified from the database of the Department of Pathology of West China Hospital, Sichuan University from January 2014 to October 2019 according to the following criteria: 1) Diagnosed with lymph node sample and fulfilled the WHO-classified diagnostic criteria of AITL (4th edition, 2008/Revised 4th edition, 2017); 2) identification of neoplastic T-cells with aberrant expression of T cell markers by flow cytometry (9); 3) identification of CD8+cells/CD4+cells > 1 by immunostaining; and 4) *de novo* cases. For comparison, we employed case-control matching to randomly choose *de novo* common AITL cases (diagnosed with lymph node sample) from a list of potential matched controls throughout the same period (2014–2019, **Supplementary Method in detail**). In order to eliminate the influence of confounding factors on the prognosis, we matched the CD8-predominant and common AITLs based on the neoplastic T-cell proportion (detected by flow cytometry) and treatment strategy. Two common AITL cases were matched to each CD8-predominant AITL case. Case-control matching was performed using SPSS (v24.0; SPSS Corp., Chicago, IL, USA). Pathological data were gathered *via* slide review by four expert hematopathologists (Z.C., S. Z., W. Z., and W. L.). The pathological analysis, flow cytometry, and the following molecular analysis were all performed on the same lymph node sample of each case. Detailed clinical data were collected from medical records. Follow-up data were obtained *via* telephone interviews and/or medical records. Overall survival (OS) was calculated from the date of diagnosis to the date of death or the last follow-up. The flowchart of case selection was provided in supplementary method (**Supplementary Material Data Sheet 1**). The study was approved by the Ethics Committee on Biomedical Research, West China Hospital of Sichuan University (No. 2021-628).

### Histological, immunohistochemical, and Epstein–Barr virus (EBV) status assessment

Sections (4 µm) were cut from paraffin blocks and stained with hematoxylin and eosin for histological examination. Immunohistochemical staining was performed using the following antibodies: cytoplasmic CD3 (cCD3), CD20, CD5, CD4, CD8, CD10, CD21, Bcl-6, CXCL13, PD1, CD30, and Ki-67. The basic information of the antibodies is summarized in the **Supplementary Method** (**Supplementary Material Data Sheet 1**). *In situ* hybridization with a digoxin-labeled oligonucleotide probe complementary to EBER-1 and EBER-2 (EBER1/2; Dako, NO. Y520001) was used to assess EBV status.

### Flow cytometry

The process for sample preparation, staining, acquisition, data analysis, and identification of neoplastic T-cells and TILs was performed in the same manner as previously described (9–11). The fluorescent-labeled antibodies used in this study are listed in **Supplementary Method** (**Supplementary Material Data Sheet 1**). Flow cytometry data were collected using the BD FACSCanto II equipped with two lasers (blue and red) and analyzed with FACSDiva software. The proportion of neoplastic T-cells and various TIL subsets, including TIL-Bs (CD20^+^), activated TIL-Bs (CD38 ^dim+^CD20^+^), non-activated TIL-Bs (CD38^-^CD20^+^), TIL-Ts (CD3^+^), CD4+TIL-Ts, CD8+TIL-Ts, and plasma cells (CD38^bight+^CD20^-^) were defined as counts of each type of cell/total counts of lymphocytes.

### TRB and IGH sequencing

DNA was isolated from formalin-fixed paraffin-embedded tissues using the QIAamp DNA FFPE Tissue Kit (Qiagen Inc., Valencia, CA, USA). For TRB and IGH repertoire sequencing, the LymphoTrack kits (TRB Assay and IGH Assay) were used following the manufacturer’s instructions with 50 ng of AITL specimen DNA as a template. Primers in the LymphoTrack assays were designed using Illumina adapters. Each amplicon was purified using AMPure XP beads (Beckman Coulter, Brea, CA, USA) and quantified using an Agilent 2100 Bioanalyzer (Santa Clara, CA, USA). Subsequently, the samples were sequenced on an Ion PGM (Thermo Fisher Scientific, Loughborough, UK). Sequencing reads were then aligned using MiXCR (v3.0.13), and only productive rearrangements were included in further analyses. Lymphoma-derived clone were identified based on TRB clonal rearrangement analysis following the manufacturer’s instructions and removed to obtain the TRB repertoire of the AITL TME (**Supplementary Method in detail**). The proportion of the top 10 clones [“% top 10 maximal frequency clones”; Frequency of each clone in AITL TME = reads of clone/(Total reads – Reads of lymphoma clone)], Shannon’s entropy (entropy), and the clonality score were calculated. The numbers of productive clones in the samples were identified by calculating the number of “productive rearrangements”. This is a measure of the number of functional T or B cells with a distinct TR or IGH rearrangement which represents the “richness.” Entropy measures both the sample richness and the degree of unevenness in clone frequencies. The clonality score describes the “evenness” of the distribution of TR or IGH clones in the repertoire, that is, how much of the TRB or IGH repertoire is composed of expanded clones independent of sample size. The top 10 clones is the frequency of the top 10 dominant clones identified in each sample. The distribution of V-gene segment usage by both TRB and IGH in CD8-predominant AITL and common AITL were compared. Both shared and differential clones with a specific complementarity determining region 3 (CDR3) sequence between the two AITL groups were identified. These analyses were performed on R using the “LymphoSeq” and “tcR” packages (12–15).

### RNA sequencing

The procedures of RNA sequencing, quality control, and normalization of data were performed by Shanghai Rightongene Biotechnology Co. Ltd (Shanghai, China), and the differentially expressed genes were identified using the R package “limma” (v3.38.3).

### Statistical analysis

Continuous variables were analyzed using the Mann–Whitney test, while categorical variables were compared using Fisher’s exact test. Survival data were calculated using the Kaplan–Meier estimator. The log-rank test was used to compare survival functions. Tests were considered significant at two-sided probability values less than 0.05 (P <0.05). All results were analyzed using GraphPad Prism (v8.0.2; GraphPad Software Inc., USA) or SPSS (v24.0; SPSS Corp., Chicago, IL, USA).

## Results

### Clinical findings

This study included six CD8-predominant AITL cases and 12 matched common AITL cases. The clinical comparison between CD8-predominant AITL and common AITL is summarized in **Table 1**. The detailed clinical features of all AITL cases included in this study are summarized in **Supplementary Table S1**. The CD8-predominant AITL cases included five men and one woman with a median age of 64.5 years (range: 48–83 years). Complete clinical data were not obtained for one patient, and the remaining five patients were classified as advanced stage (stage III/IV) with frequent splenomegaly (5/5, 100%), B symptoms (4/5, 80%), and hepatomegaly (3/5, 60%). In general, these baseline features were comparable to the matched common AITL cases. Edema (6/6,100%, *P =* 0.011) and serous effusion (5/5,100%, *P =* 0.026) were more prevalent in CD8-predominant AITL than in common AITL cases. In laboratory studies, CD8-predominant AITL exhibited findings identical to those of common AITL cases in routine blood tests and immunological assays. While lower fibrinogen (2.09 g/L, range: 1.60–2.56 g/L, *P =* 0.038) and more common decreased C4 (3/3, 100%, *P =* 0.033) were detected in CD8-predominant AITL than in common AITL cases. Highly elevated plasma EBV-DNA (> 10^4^ copies/ml) was detected in all tested CD8-predominant AITL (3/3, 100%) but not in any common AITL cases (0/7, *P =* 0.008).

**Table 1:** Clinical comparison between microenvironment CD8-predominant AITLs and matched normal AITLs.

	CD8-predominant AITL	Common AITL	*P*
**Age [median, (range)]**	64.5y(48y-83y)	59y(43y-82y)	0.483
**Sex (male/female)**	5/1	7/5	0.600
**Stage**			1.000
** I/II**	0 (0)	1 (17)	
** III/IV**	5 (100)	5 (83)	
**PS ≥2**	2 (40)	3 (27)	1.000
**B symptoms**	4 (80)	7 (58)	0.600
**Fever**	3 (60)	5 (42)	0.620
**Night sweat**	0 (0)	3(25)	0.515
**Weight loss**	1 (20)	4 (33)	1.000
**Skin rash**	1 (20)	5 (45)	0.588
**Edema**	6 (100)	3 (30)	***0.011***
**Serous effusion**	5 (100)	3 (30)	***0.026***
**Splenomegaly**	5 (100)	4 (44)	0.086
**Hepatomegaly**	3 (60)	1 (11)	0.095
**Anemia**	3 (60)	7 (64)	1.000
**Thrombocytopenia**	2 (40)	0 (0)	0.083
**Leukocytosis**	3(60)	1 (9)	0.063
**EO (×10^9^/L) [median, (range)]**	0.22 (0.02-5.86)	0.25 (0.01-1.16)	0.827
**FIB (g/L) [median, (range)]**	2.09 (1.60-2.56)	3.08 (1.84-6.79)	***0.038***
**Elevated LDH**	3 (75)	5 (71)	1.000
**CD4+T-cells in PB (%)** **[median, (range)]**	23.90 (12.00-32.60)	25.2 (19.70-36.30)	1.000
**CD8+T-cells in PB (%)** **[median, (range)]**	48.00 (33.40-57.00)	31.00 (18.50-40.70)	0.111
**Elevated IgG**	2 (50)	4 (57)	1.000
**Elevated IgA**	2 (50)	4 (57)	1.000
**Abnormal IgM**	2 (50)	3 (43)	1.000
**Elevated IgE**	2 (67)	2 (33)	0.524
**Decreased C3**	3 (100)	3 (43)	0.200
**Decreased C4**	3 (100)	1 (14)	***0.033***
**High plasma EBV-DNA** **(>10^4^ copies/ml)**	3 (100)	0 (0)	***0.008***
**Chemotherapy**	3 (50)	6 (50)	1.000

PS, performance status; EO, Eosinophils; FIB, Fibrinogen; PB, peripheral blood. Bold value indicates statistically significant (P < 0.05).

### Pathological findings

**Figure 1:**
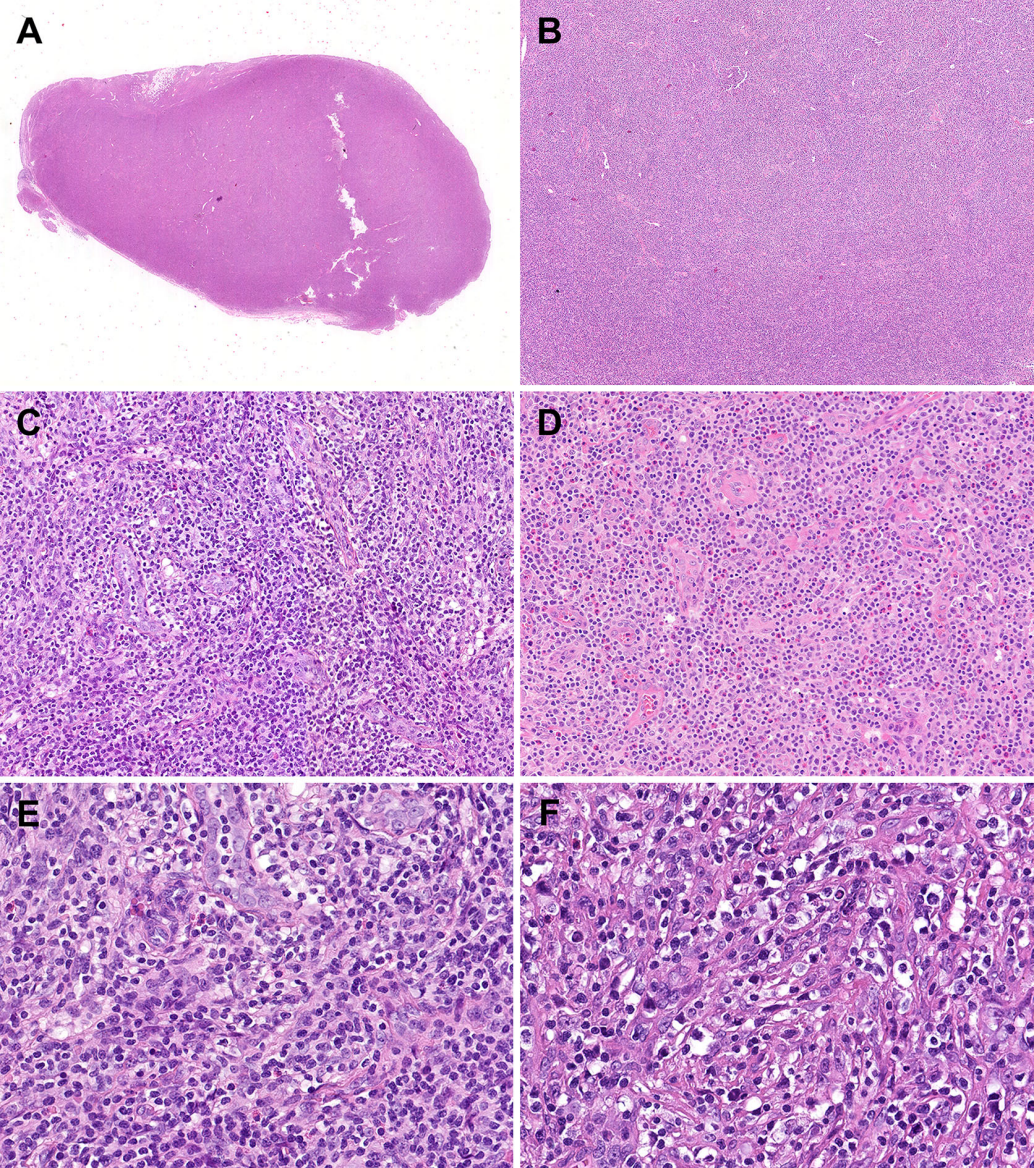
Morphological features of CD8-predominant AITL. In the hematoxylin & eosin staining, the CD8-predominant AITL exhibited **(A)** effaced lymph node architecture (×10), **(B)** in a diffuse growth pattern (×40), **(C)** with arborizing HEV proliferation (×200) and **(D)** eosinophil increase (×200). **(E)** Medium-sized (×400) or **(F)** medium-large-sized (×400) neoplastic T-cells with clear to pale cytoplasm surrounding HEVs.

**Figure 2:**
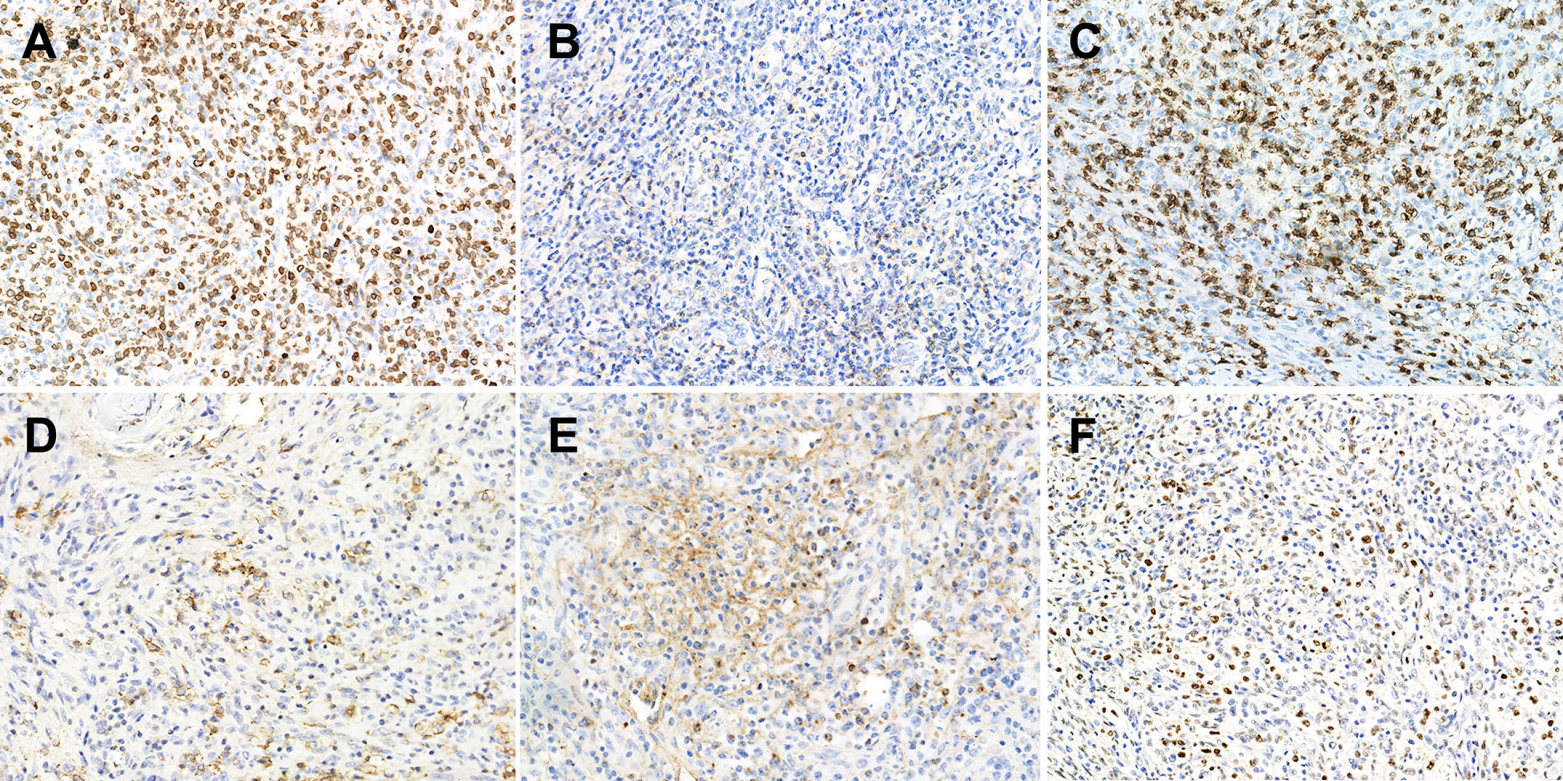
Immunophenotype of CD8-predominant AITL. **(A)** CD3 (×200); **(B)** CD4 (×200); **(C)** CD8 (×200); **(D)** PD1 (×200); **(E)** CXCL13 (×200); **(F)** Bcl-6 (×200).

Histopathological comparisons between CD8-predominant AITLs and matched common AITLs are summarized in **Table 2**. In general, CD8-predominant AITL had characteristic AITL histological features, with effaced lymph node architecture (6/6, 100%, **Figure 1A**) in a diffuse growth pattern (6/6, 100%, **Figure 1B**) harboring arborizing proliferation of HEVs (6/6, 100%, **Figure 1C**). In two cases, the proliferating cells were medium-sized (**Figure 1E**), and in four cases, they were medium-large sized (**Figure 1F**). These morphological findings were consistent with those found in the matched common AITL cases. Nonetheless, CD8-predominant AITL exhibited more significant eosinophil increase [eosinophil cell count > 30/high power filed (HPF), 5/6, 83%, **Figure 1D**] than common AITL cases (1/12, 8%, *P =* 0.004), which is a distinguishing morphological feature. Immunohistochemically, CD8-predominant AITL was diffusely positive for CD3 (**Figure 2A**) and ≥3 TFH markers (**Figures 2D-F**). CD8+cells outnumbered CD4+cells (**Figures 2B, C**). CD8-predominant AITL exhibited a higher CD30 positive rate (median: 20%, range: 0–30%, *P =* 0.020) and Ki67 index (median: 60%, range: 50–70%, *P =* 0.041) than common AITL. All CD8-predominant AITL cases tested positive for EBER with a median positive cell count of 7.5/HPF (range: 1–150/HPF), which is higher than in common AITL (median: 3.5/HPF, range: 0–20/HFP), although not statistically significant (*P =* 0.263).

**Table 2:** Pathological comparison between CD8-predominant AITLs and matched normal AITLs.

	CD8-predominant AITL	Common AITL	*P*
**Effaced LN architecture**	6 (100)	9 (75)	0.515
**Diffuse growth pattern**	6 (100)	9 (75)	0.515
**Arborizing proliferation of HEVs**	6 (100)	12 (100)	1.000
**Cell size**			0.254
** Small**	0 (0)	0 (0)	
** Small-Medium**	0 (0)	2 (16)	
** Medium**	2 (33)	5 (42)	
** Medium-Large**	4 (67)	5 (42)	
** Large**	0 (0)	0(0)	
**EO increase (>30/HPF)**	5 (83)	1 (8)	***0.004***
**TFH cell marker**			0.686
** 2 markers**	0 (0)	1 (8)	
** 3 markers**	4 (67)	5 (42)	
** 4 markers**	2 (33)	6(50)	
**CD30 (%)**	20 (0-30)	5 (0-15)	***0.020***
**Ki67 (%)**	60 (50-70)	50 (15-80)	***0.041***
**EBER/HPF**	7.5 (1-150)	3.5 (0-20)	0.263

TFH, T follicular helper; LN, lymph node; EO, eosinophil; HEVs, high endothelial venules; HPF, high power filed. Bold value indicates statistically significant (P < 0.05).

### Flow cytometry evaluated neoplastic T-cell and TIL subset proportions in CD8-predominant AITLs and common AITLs

**Figure 3:**
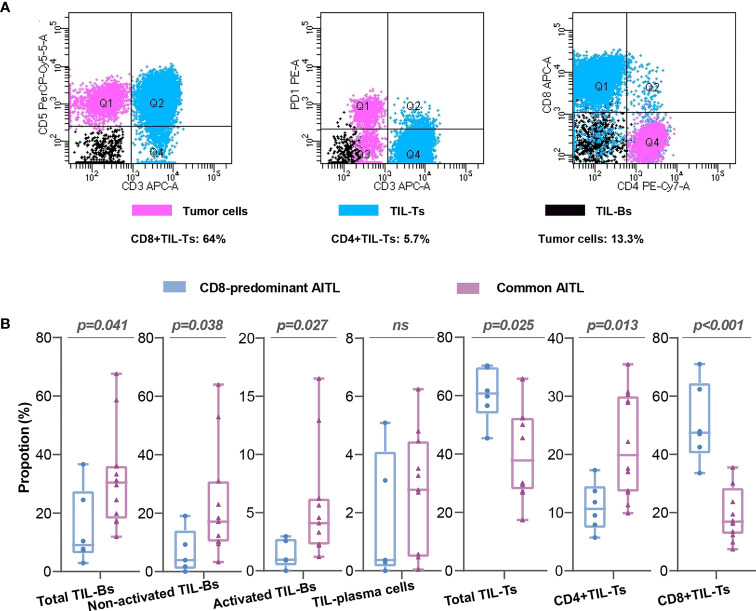
Flow cytometry analysis of CD8-predominant AITLs and matched common AITLs. Flow cytometry analysis of a typical case (C1) of CD8-predominant AITL **(A)** Comparisons of TIL subsets in flow cytometry analysis between CD8-predominant AITLs and matched common AITLs **(B)**. “ns” indicates “no significance”.

The neoplastic T-cells in CD8-predominant AITLs had a median proportion of 13% (neoplastic T-cells/total lymphocytes; range: 3.5–21.8%, **Table S1**). The CD4+TIL-Ts/CD8+TIL-Ts was ranged from 0.09 to 0.41 (**Table S1**). A typical case (C1) is shown in **Figure 3A**. The included common AITLs had matched tumor cell proportions with a median of 12.1% (range: 4.3–23.5%, **Table S1**).

TIL subset proportions in CD8-predominant AITLs differed from those in common AITLs. CD8-predominant AITLs had a significantly higher proportion of total TIL-Ts (CD8-predominant AITL: median: 60.8%, range: 45.4–70.3%, vs. common AITL: median: 37.8%, range: 17.4–65.8%, P = 0.025), CD8+TIL-Ts (CD8-predominant AITL: median: 47.5%, range: 33.6–64.0%, vs. common AITL: median: 16.9%, range: 7.4–35.5%, p < 0.001), and lower CD4+TIL-Ts (CD8-predominant AITL: median: 10.7%, range: 5.7–17.3%, vs. common AITL: median: 19.9%, range: 9.9–35.5%, P = 0.013). Furthermore, total TIL-Bs (median: 9.1%, range: 2.9–36.7%), activated TIL-Bs (median: 0.9%, range: 0–2.9%), and non-activated TIL-Bs (median: 3.9%, range: 0–19.0%) had lower proportions in CD8-predominant AITLs than in common AITLs (total TIL-Bs, median: 30.4%, range: 11.9–67.6%, p=0.041; activated TIL-Bs, median: 4.1%, range: 1.2–16.5%, P = 0.027; non-activated TIL-Bs, median: 17.1%, range: 3.3–64.0%, P = 0.038). However, there was no significant difference between CD8-predominant and common AITLs in the proportion of TIL-plasma cells. **Figure 3B** depicts the TIL subset comparisons in flow cytometry analysis.

### TRB repertoires

**Figure 4:**
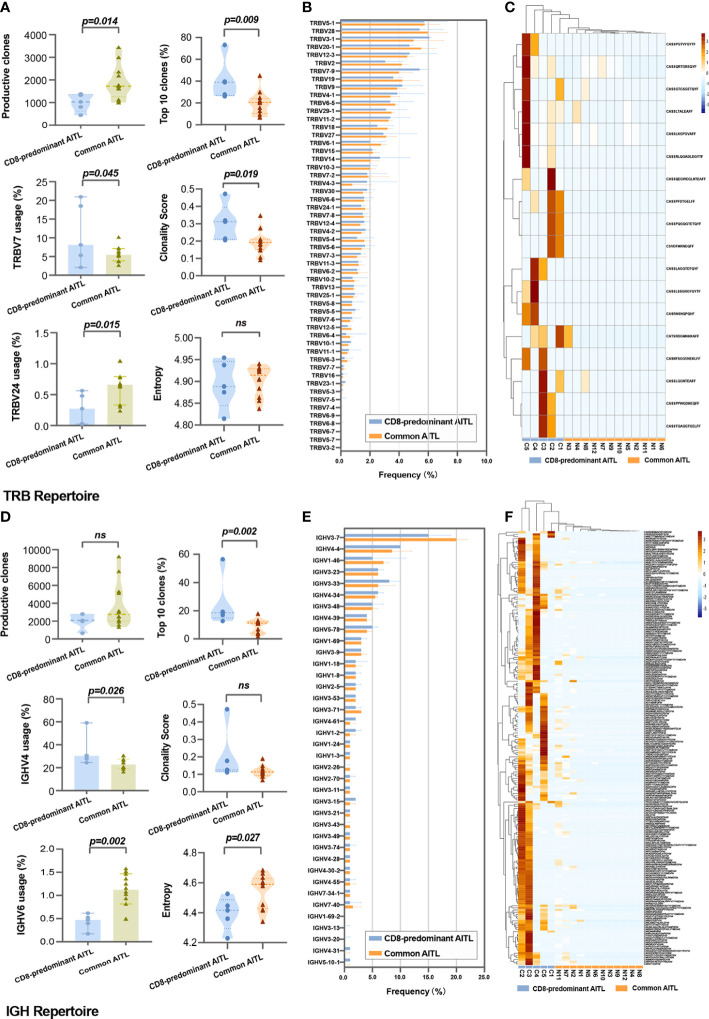
Comparison of TRB and IGH repertoires between CD8-predominant AITLs and matched common AITLs. Comparison between two AITL groups TME in statistical metrics including production clones, V-gene family usage, top 10 clones’ proportion, clonality score, and entropy of TRB repertoire **(A)** and IGH repertoire **(D)**, in global usage of V-gene of TRB repertoire **(B)** and IGH repertoire **(E)**, and in the CDR3 sequence frequency of the TRB **(C)** and IGH repertoires **(F)**. “ns” indicates “no significance”.

TRB sequencing was performed on five of six patients with CD8-predominant AITL and all 12 patients with common AITL. For the TRB repertoire in the TME, CD8-predominant AITLs exhibited significantly lower productive clones (median: 998, range: 443–1378) than those of common AITLs (median: 1727, range: 997–3444, P = 0.014, **Figure 4A**). The CD8-predominant AITLs had a significantly higher proportion of the top 10 clones [CD8-predominant AITLs median top 10 clones’ proportion of 39.1% (range 26.1–73.2%) vs. common AITL median top 10 clones’ proportion of 20.6% (range 6.9–45.1%), P = 0.009], and higher clonality score [CD8-predominant AITLs median clonality score 0.312 (range 0.205–0.472) vs. common AITL median clonality score 0.191 (range 0.091 –0. 347), P = 0.019]. The entropy did not differ between the two groups (**Figure 4A**).

The global usage of TRBV segments in the TRB repertoire was not different between the two AITL groups (**Figure 4B**). However, we found significantly increased usage of the TRBV7 family segment (median: 8.8% vs. 5.5%, P = 0.045) and lower usage of the TRBV24 family (median: 0.3% vs. 0.7%%, P = 0.015) in CD8-predominant AITLs than in common AITLs (**Figure 4A**). A shared clone with the “CASSFSTCSANYGYTF” CDR3 sequence was identified in all 17 tested AITLs. Furthermore, we found that 18 CDR3 sequences were more frequently identified in CD8-predominant AITLs than in common AITLs (p<0.05, **Figure 4C**). All shared and differential TRB CDR3 data are provided in **Supplementary Table S2**.

### IGH repertoires

IGH sequencing was performed on the same samples as the TRB sequencing. The productive clones were not different between CD8-predominant AITLs and common AITLs (**Figure 4D**). CD8-predominant AITLs had a higher proportion of the top 10 clones [CD8-predominant AITLs top 10 clones, 18.5% (range 12.7–56.5%) vs. common AITL top 10 clones, 11.1% (range 1.9–17.9%), P = 0.002], lower entropy [CD8-predominant AITLs median entropy 4.415 (range 4.229–4.526) vs. common AITL median entropy 4.589 (range 4.341–4.684), P = 0.027]. The clonality score did not differ between the two groups (**Figure 4D**).

The global usage of IGHV segments did not differ between CD8-predominant AITLs and common AITLs, with IGHV3-7 being the most frequently used segment in both AITL groups (**Figure 4E**). Then, CD8-predominant AITL utilized the IGHV4 family segment more frequently (median: 30.3% vs. 22.7%, P = 0.026) and utilized the IGHV6 family segment less frequently (median: 0.5% vs. 1.1%, P = 0.002, **Figure 4D**) than common AITLs. We also found a shared IGH CDR3 sequence of “CARPYSYGYGDYVYYYGMDVW” in all tested AITL cases, which was identified more frequently in CD8-predominant AITLs (median: 10.4%, range: 1.1% - 48.9%) than in common AITLs (median: 0.9%, range: 0.01% - 8.6%, P = 0.027). Moreover, we discovered 303 IGH CDR3 sequences that differed in frequency between the two AITL groups (**Figure 4F** displayed differential IGH CDR3 with p < 0.033, n = 201). **Supplementary Table S3** contains all the shared and differential IGH CDR3 sequences.

### Comparative gene expression profiling between CD8-predominant and common AITL for inflammation and immune response

**Figure 5:**
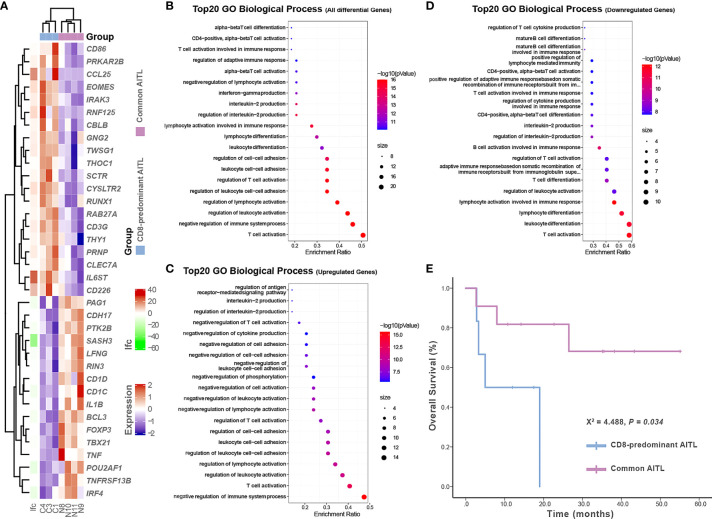
Gene expression profiles and survival analysis in CD8-predominant AITLs and matched common AITLs. Heatmap analysis showed differences in inflammation and immune response-related gene expression between CD8-predominant AITLs and common AITLs **(A)**. The top 20 enriched biological processes in gene oncology from all differential genes **(B)**, upregulated differential genes **(C)**, and downregulated differential genes **(D)**. Kaplan–Meier estimates of overall survival rate for CD8-predominant AITL and matched patients with common AITL **(E)**.

RNA sequencing was performed on three CD8-predominant AITLs and four common AITLs. To compare these two groups for inflammation and immune response, we created a panel of 772 genes based on the gene ontology biological process annotation (**Supplementary Method** in detail, the gene expression profile data were provided in **Supplementary Table S4**). Comparative analysis identified 36 genes in this panel with significant differential expression between CD8-predominant AITL and common AITL through the linear model fit with p-value correction (Padj < 0.05 and log2fc > 1), of which 20 genes were upregulated and 16 genes were downregulated in CD8-predominant AITLs (**Figure 5A**). Functional annotation analysis revealed predominant enriched differentially expressed genes in T-cell activation, negative regulation of immune system process, regulation of leukocyte activation, and regulation of lymphocyte activation (**Figure 5B**). CD8-predominant AITL was considerably enriched for upregulated negative regulation of immune system processes (**Figure 5C**) and downregulated T-cell activation and immune cell differentiation (**Figure 5D**) compared with common AITL, which suggested suppressed immune function.

### Survival analysis

Survival data were obtained from all six patients with CD8-predominant AITL and 11 of the 12 patients with common AITL. Four patients with CD8-predominant AITL (4/6, 67%) and three patients with common AITL (3/11, 27%) died of the disease. Besides tumor proportion and treatment strategy, the other recognized prognostic factors were also comparable between the two AITL groups (**Table 1**). The prognosis of patients with CD8-predominant AITL (median overall survival: 5.0 months, 95%CI: 0–14.4 months) was significantly worse than that for those with common AITL (median overall survival not reached, P = 0.034, **Figure 5E**).

## Discussion

AITL is highly heterogeneous, and exhibits complex TMEs (16). Our previous research has discovered that the ratio of the components, particularly TILs, varies in AITL cases (9). When CD8+TIL-Ts proliferated abundantly, they outnumbered CD4+ cells, formatting the CD8-predominant AITL. Because TME is directly associated with immune response, investigating TME immune activity in CD8-predominant AITL may facilitate immune-subtyping and prognostic stratification of AITL cases. We conducted an integrated analysis of the TME of CD8-predominant AITLs and compared them to common AITLs. To reduce the impact of tumor cell proportion on the microenvironment and treatment effect on prognosis, we matched these features in two groups of AITL cases.

To compare TME functions between the two AITL groups, TIL subset proportion, TRB and IGH repertoires, and gene expression profiles were examined. TIL subsets and proportions directly reflected TME immune function and have been shown to be predictive of various malignancies (17–19). TR and IGH repertoires of TME dictate TIL-T and TIL-B variety and represent the ability to identify tumor antigens and combat tumor immunoevasion (13, 14). Furthermore, gene expression profiling may reveal immunological activity by examining the regulation of related pathways. Our findings revealed that CD8-predominant AITL differs significantly from common AITL, which appears to have an immunosuppressive TME.

Although CD8-predominant AITL shared histopathological characteristics with common AITL in diffuse growth patterns and arborizing HEVs, it showed an increase in eosinophil counts. High eosinophil counts have been reported in AITL and have been described to be associated with cytotoxic agents, which may partly imply the cause of our findings (20). Meanwhile, CD8-predominant AITL showed in CD8+ TIL-Ts and a decrease in both CD4+TIL-Ts and TIL-Bs, implying a dysimmune TME. Despite the increase of CD8+TIL-Ts in CD8-predominant AITL, single-cell transcriptome analysis of AITL TME revealed that CD8+TIL-Ts expressed CD45RO, CD27, PD1, and TIGIT, indicating an exhausted phenotype (21). Therefore, CD8-predominant AITL may have reduced cytotoxicity in the TME. Furthermore, CD4+TIL-Ts are required for CD8+ TIL-T activation, and their depletion may result in CD8+ TIL-T impotence (22, 23). Moreover, CD4+TIL-Ts exert anti-tumor effects by recognizing tumor antigens and releasing cytokines, which activate other tumoricidal immune cells (24). Therefore, the low level of CD4+TIL-Ts in CD8-predominant AITL also indicated that anti-tumor immunity was downregulated. Additionally, neoplastic T cells in AITL still preserve the function of TFH cells that secrete CXCL13 and other cytokines to promote B cell recruitment, expansion, and activation, generally resulting in a B-cell-rich TME (6, 7, 25, 26). Moreover, our previous study found that the expanded B-cells represent an efficient humoral response and enhance cellular immunity through T-cell and B-cell crosstalk in the TME, leading to good survival (9, 27). As a result, the lower TIL-Bs in CD8-predominant AITL implied suppressed humoral response and immune dysfunction in the TME.

CD8-predominant AITLs displayed narrowed TRB and IGH repertoires in TME, showing a significant drop in productive clones and increased clonality score in the TRB repertoire, a decrease in entropy, and an increase in the proportion of the top 10 clones in the IGH repertoire, which also indicated the immunosuppressive TME and anti-tumor immunity impairment. TR and IGH repertoires, the essential determinants of the TME in both solid tumors and hematological malignancies, reflect the immune activity of the TME (12, 15). Previous investigations have shown that deteriorated TR repertoire metrics could be caused by immunosuppression due to aging or inborn errors (28–31). Accordingly, our findings in the TRB repertoire of the CD8-predominant AITL TME may be interpreted by TME immunosuppression. Additionally, in keeping with knowledge from other tumors (13, 32), the narrowed TR and IGH repertoires in the TME of CD8-predominant AITL may indicate downregulated anti-tumor immunity *via* inadequate immunosurveillance of tumor T-cell neoantigens.

The gene expression profile further supported the findings of flow cytometry analysis and TRB and IGH repertoire sequencing. RNA sequencing data were analyzed focusing on genes related to inflammation and immune response, revealing the increased negative regulation of immune system processes and decreased T-cell activation and immune cell differentiation compared to common AITL. These results directly demonstrate the immunosuppressive TME in CD8-predominant AITL.

Neoplastic T-cells in AITL shape the TME in terms of histological structure and immune pattern by secreting cytokines and producing tumor antigens (7, 33). Therefore, TME similarity is closely related to tumor cell similarity. Neoplastic AITL cells retain TFH cell functions and harbor a recurrent RHOA**^G17V^** mutation (6). Furthermore, in our previous study, AITL tumor cells were found to have a biased usage of TRBV19-1 and a high level of identity in the CDR3 sequence (34). Thus, these commonalities of AITL tumor cells could explain our findings: CD8-predominant AITLs and common AITLs had similar histological structures and shared CDR3 sequences in the TME TRB and IGH repertoires. Neoplastic cell heterogeneity is associated with differences in the TME. We found that CD8-predominant AITLs and common AITLs had different V family segment usage and CDR3 sequence frequency in the TME of both TRB and IGH repertoires. Consistent with the literature on solid tumors and hematopoietic malignancies, these findings reflect the heterogeneity of the tumor antigen portfolio and further imply a heterogeneous mutational landscape of neoplastic cells between the two AITL groups (35–37). Thus, heterogeneity of neoplastic T-cells may be the cause of TME immune function differences between the two AITL groups, resulting in CD8-predominant AITL having an immunosuppressive TME.

**Figure 6:**
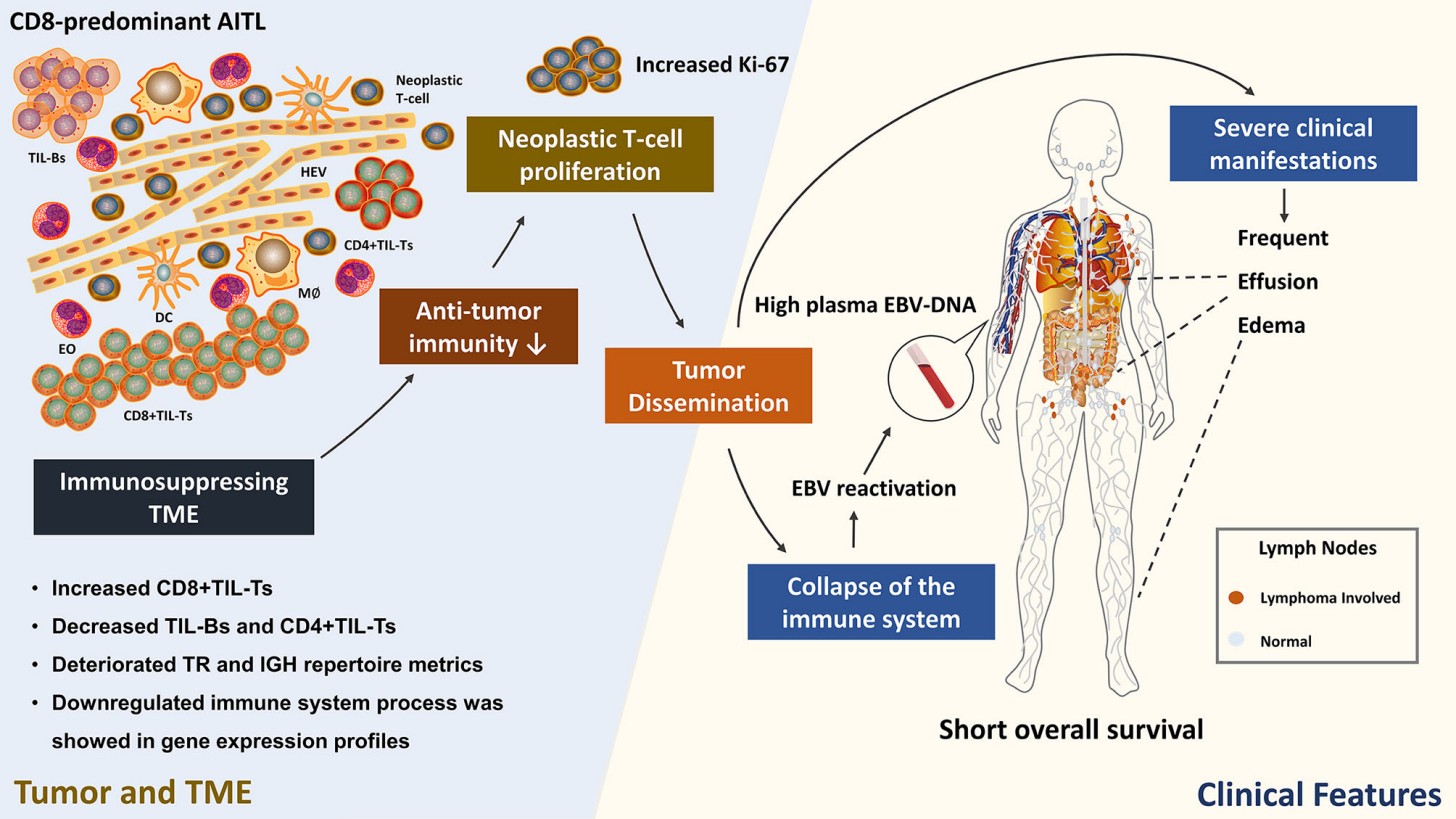
Hypothesized correlation between CD8-predominant AITL TME immune function and clinicopathological features. TIL, tumor infiltrating lymphocyte; HEV, high endothelial venule; EO, eosinophil; DC, dendritic cell; M∅, macrophage. This figure was drawn using ScienceSlides 2016, VisiScience, Inc.

Studies from both lymphoma and other solid tumors demonstrated that the TME changes as tumor progresses; in general, the immune response of the TME gradually decreases and eventually leads to immune silence (38, 39). Our results demonstrated that CD8-predominant AITL has an immunosuppressive TME, indicating that CD8-predominant AITL is at a later stage of tumor development and thus has significantly inferior survival compared to common AITLs. Combined with the patient’s clinical manifestations, we hypothesized that the immunosuppressed TME of CD8-predominant AITL leads to insufficient anti-tumor immunity, manifesting as an increase in tumor proliferation index, which in turn leads to tumor dissemination (40), causing aggravation of the patient’s clinical manifestations such as frequent effusion and edema. Furthermore, the patients’ immune system collapses secondary to tumor development, resulting in EBV reactivation and increased plasma EBV-DNA (41). This eventually leads to a reduction in the patient’s survival (**Figure 6**). Therefore, future research should concentrate on enhancing the immune function of the TME of CD8-predominant AITL, thereby improving patient prognosis.

This study has potential limitations. The main point is the small sample size, which is due to the rarity of CD8-predominant AITL. To reduce the confounding factors and sampling error, we introduced the case-control matching. Nevertheless, our findings must be validated through the accumulation of cases.

## Conclusions

CD8-predominant AITL is an uncommon disease with more severe clinical manifestations and a shorter OS than that of common AITL. Although CD8-predominant AITL has histological features similar to those of common AITL, it has a lower TIL-B proportion, an inverted CD4+/CD8+ TIL-Ts ratio, deterioration of TR and IGH repertoire metrics, and altered gene expression profiles, indicating the anti-tumor immunity impairment in TME. According to the accumulated evidence, CD8-predominant AITL is a distinct immune pattern of AITL with an immunosuppressive TME that must be better identified and investigated to improve patient survival.

## Data availability statement

TRB and IGH sequencing data have been deposited in the National Center for Biotechnology Information Sequence Read Archive under accession number PRJNA887199. The RNA-seq data were provided in the **Supplementary Material**.

## Ethics statement

The studies involving human participants were reviewed and approved by Ethics Committee on Biomedical Research, West China Hospital of Sichuan University. Written informed consent for participation was not required for this study in accordance with the national legislation and the institutional requirements.

## Author contributions

ZC, YT, and SZ designed and conceived the study. ZC, QZ, WZ, WL, and SZ selected samples. ZC, QZ, and XD performed experiments. ZC, QZ, and WY analyzed the data. ZC wrote the manuscript. All authors contributed to the article and approved the submitted version.

## Funding

This study was supported by the National Natural Science Foundation of China (81900195, 30900534), 1·3·5 project for disciplines of excellence–Clinical Research Incubation Project, West China Hospital, Sichuan University (2021HXFH027), and Post-Doctor Research Project, West China Hospital, Sichuan University (2019HXBH067).

## Conflict of interest

The authors declare that the research was conducted in the absence of any commercial or financial relationships that could be construed as a potential conflict of interest.

## Publisher’s note

All claims expressed in this article are solely those of the authors and do not necessarily represent those of their affiliated organizations, or those of the publisher, the editors and the reviewers. Any product that may be evaluated in this article, or claim that may be made by its manufacturer, is not guaranteed or endorsed by the publisher.
