# Supplementary material for: Angioimmunoblastic T-cell lymphoma with predominant CD8+ tumor-infiltrating T-cells is a distinct immune pattern with an immunosuppressive microenvironment
Source: Front Immunol. 2022 Oct 17;13:987227. doi: 10.3389/fimmu.2022.987227 (PMC9618886; doi:10.3389/fimmu.2022.987227)
Supplement: Supplementary file 1 [file DataSheet_1.docx]

**Supplementary method**

***Case Selection***

Flowchart of case selection


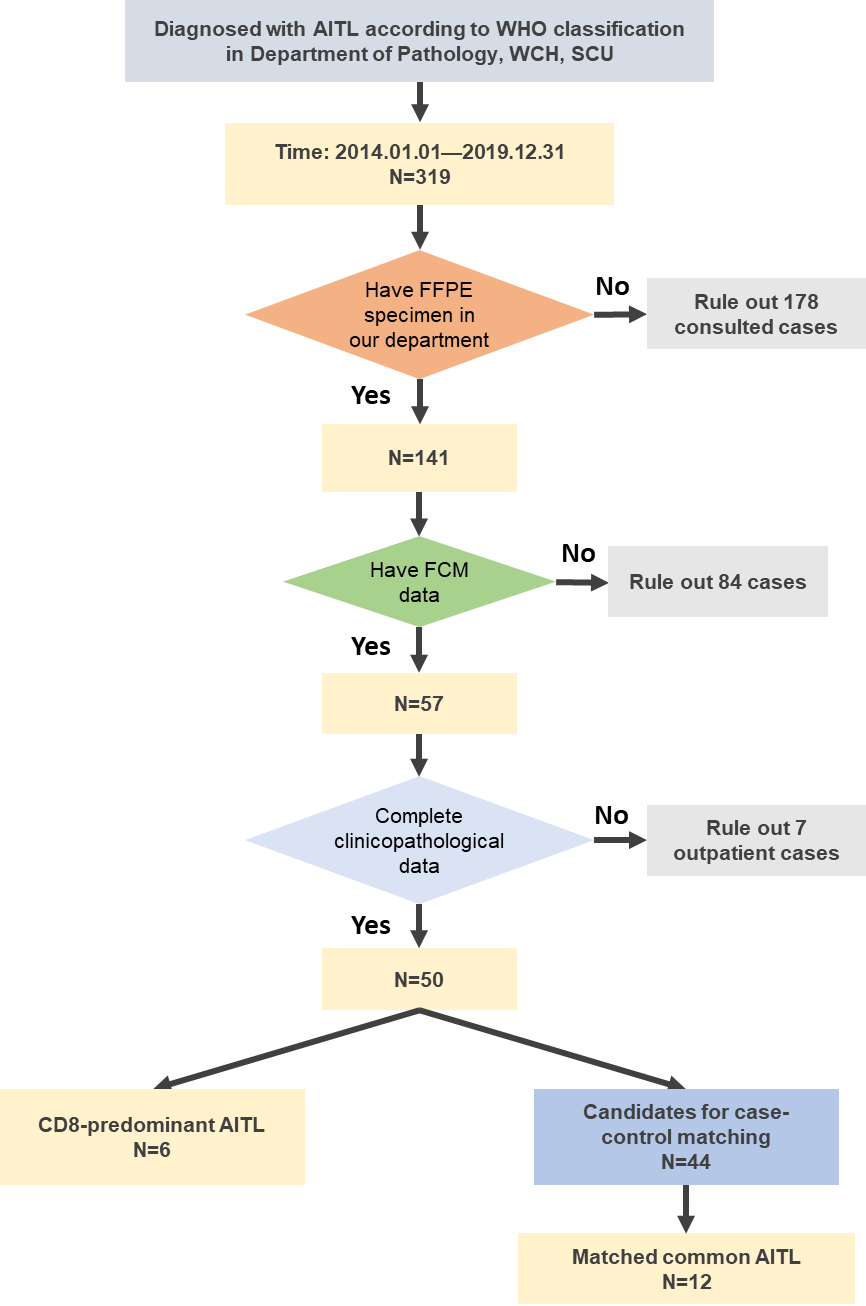


***Histological, Immunohistochemical, and Epstein–Barr virus (EBV) Status Assessment***

Antibody reagents for immunohistochemical stating in this study

| **Antibody** | **Clonal** | **Manufacturer** | **Dilution** |
| --- | --- | --- | --- |
| CD20 | L26 | DAKO | 1:100 |
| CD2 | 2CO2 | Neomarkers | 1:100 |
| cCD3 | PS1 | DAKO | 1:100 |
| CD4 | 1F6 | Maixin | 1:50 |
| CD5 | 4C7 | Novocastra | 1:100 |
| CD8 | C8 | Maixin | 1:100 |
| CD10 | SP67 | ZhongShan | 1:150 |
| CD21 | EP64 | MaiXin | 1:100 |
| CD30 | Ber-H2 | Neomarkers | 1:50 |
| Bcl-6 | MX042 | MaiXin | 1:100 |
| CXCL13 | polyclonal | ZhongShan | 1:100 |
| PD1 | UMAB199 | ZhongShan | 1:50 |
| βF1 | TCR1151 | Endogen | 1:100 |
| Ki-67 | MIB1 | Neomarkers | 1:150 |

***Flow Cytometry***

Antibody reagents for flow cytometry in this study

| **Antibody** | **clonal** | **Fluorescence** | **Manufacturer** |
| --- | --- | --- | --- |
| CD2 | S5.2 | FITC | BD |
| CD3 | SK7 | PE/Percp-cy5-5 | BD |
| CD4 | SK3 | PE-cy7 | BD |
| CD5 | L17F12 | PerCP-cy5-5 | BD |
| CD7 | 4H9 | PE | BD |
| CD8 | SK1 | APC | BD |
| CD19 | SJ25C1 | AmCyam | BD |
| CD20 | L27 | APC-cy7 | BD |
| CD30 | Ber-H83 | FITC | BD |
| CD38 | HB7 | APC | BD |
| CD45 | 2D1 | APC-cy7 | BD |
| CD56 | NCAM16.2 | PE-cy7 | BD |
| Kappa | TB28-2 | FITC | BD |
| Lambda | 1-155-2 | PE | BD |

***TRB and IGH Sequencing***

According to the manufacturer’s instructions, TRB clonal rearrangement (monoclonal TRB rearrangement) was defined as follow and Top 1 clone in the case with monoclonal TRB rearrangement was regarded as lymphoma-derived clone

| **Condition** | **Criteria for monoclonal TRB rearrangement** |
| --- | --- |
| Total reads ≥20,000 | Reads of Top 1 clone > 2.5% total reads  Reads of Top 1 clone > twice of the reads of Top 3 clone |
| 10,000≤ Total reads <20,000 | Reads of Top 1 clone > 5% total reads  Reads of Top 1 clone > twice of the reads of Top 3 clone |

.

The brief information of TRB sequencing data and identified lymphoma-derived clone of each case in this study were listed as follow:

|  |  | **Lymphoma derived clone** | | |
| --- | --- | --- | --- | --- |
| **Case** | **Total reads** | **Reads** | **Frequency**  **(%)** | **CDR3** |
| C1 | 80960 | 9213 | 11.4 | CASSLGTGTNTEAFF |
| C2 | 81163 | 22837 | 25.1 | CASSRPQGEDTEAFF |
| C3 | 292931 | 101940 | 34.8 | CASSLLLGTEAFF |
| C4 | 270339 | 45417 | 16.8 | CATRYNYGYTF |
| C5 | 14544 | 2647 | 19.2 | CASSQETGRSDPLHF |
| N1 | 48638 | 12784 | 26.3 | CASSTANTEAFF |
| N2 | 145954 | 49008 | 33.8 | CASSFNQTPPLCYEQYF |
| N3 | 175029 | 50651 | 28.9 | CSAVGSYNEQFF |
| N4 | 248657 | 51822 | 18.3 | CSARYGTSGYNEQFF |
| N5 | 315572 | 163151 | 51.7 | CASSQEGWEGLNTEAFF |
| N6 | 173384 | 28790 | 16.6 | CASSLGRRVLEAFF |
| N7 | 117206 | 45876 | 39.1 | CASSATGDQPQHF |
| N8 | 222165 | 41545 | 18.7 | CASSQLETQYF |
| N9 | 96463 | 17074 | 17.7 | CASSRPGDYGYTF |
| N10 | 227583 | 36641 | 16.1 | CASSRQGTGDTQYF |
| N11 | 169391 | 11681 | 6.9 | CASSQVSTSGETQYF |
| N12 | 207255 | 95575 | 46.1 | CASSYSGGTYGYTF |

***RNA Sequencing***

The gene expression analysis was focus on the inflammation and immune response. The gene panel (772 genes) was designed based on the gene ontology biological process annotation. To reduce selection bias, all genes related to inflammation and immune response in the database of gene ontology biological process annotation (http://amigo.geneontology.org/amigo/search/annotation) were obtained and then the duplication, non-human genes, and gene products were removed. Then, in this designed gene panel, a comparative analysis was performed to identify the significantly differentially expressed genes. Finally, functional annotation was performed on the significantly differentially expressed genes.

Inflammation and immune response related gene panel

| ***TAG*** | ***BCL3*** | ***EOMES*** | ***CYSLTR2*** | ***SASH3*** | ***CD3G*** | ***RAB27A*** | ***TNFRSF13B*** | ***CDH17*** | ***TBX21*** |
| --- | --- | --- | --- | --- | --- | --- | --- | --- | --- |
| ***FOXP3*** | ***IRAK3*** | ***PTK2B*** | ***IL6ST*** | ***SCTR*** | ***RIN3*** | ***PRKAR2B*** | ***SLIT2*** | ***TWSG1*** | ***DLG5*** |
| ***PAG1*** | ***LFNG*** | ***PRNP*** | ***TRIM27*** | ***RNF125*** | ***RUNX1*** | ***CBLB*** | ***IL1B*** | ***FAM3A*** | ***SOCS6*** |
| ***CD1C*** | ***POU2AF1*** | ***INHA*** | ***CLEC7A*** | ***TNF*** | ***GNG2*** | ***CD86*** | ***THOC1*** | ***CCL25*** | ***CD1D*** |
| ***CD226*** | ***IRF4*** | ***THY1*** | ***OTOP1*** | ***KLRK1*** | ***RAMP1*** | ***C3*** | ***DTX1*** | ***GPR27*** | ***RELB*** |
| ***CRH*** | ***P2RY11*** | ***HAVCR2*** | ***GPR116*** | ***IL4*** | ***SYK*** | ***ITFG2*** | ***PRKCQ*** | ***MERTK*** | ***ALOX15*** |
| ***TLR4*** | ***HMCES*** | ***SFTPD*** | ***HMGB1*** | ***ZBTB1*** | ***DPP4*** | ***FZD5*** | ***DUSP3*** | ***GNRH1*** | ***NFKBID*** |
| ***DOCK11*** | ***SAMHD1*** | ***OAS3*** | ***IL1RL1*** | ***HTRA1*** | ***NFKBIZ*** | ***CD163*** | ***IL18*** | ***IL13RA2*** | ***CD40LG*** |
| ***CORO1A*** | ***LEF1*** | ***PTPN6*** | ***NLRC3*** | ***ANGPT1*** | ***PDCD1LG2*** | ***JAK3*** | ***IFI16*** | ***MAFB*** | ***SRC*** |
| ***NR1D1*** | ***TRAFD1*** | ***TOB2*** | ***SERPINB9*** | ***RSAD2*** | ***RARA*** | ***IL1R1*** | ***ZBTB7B*** | ***WASL*** | ***FAM65B*** |
| ***CD1A*** | ***ADCY1*** | ***PIBF1*** | ***IL10*** | ***CLEC4G*** | ***SYT11*** | ***TMEM98*** | ***LGALS9C*** | ***LOXL3*** | ***PPP3CB*** |
| ***ZFPM1*** | ***GNB1*** | ***C1QC*** | ***NME1*** | ***CD200R1*** | ***ADRB2*** | ***MC4R*** | ***ADORA2A*** | ***FBXO7*** | ***TSPAN32*** |
| ***KITLG*** | ***CACTIN*** | ***RHBDF2*** | ***GPATCH3*** | ***CD19*** | ***MFNG*** | ***PCBP2*** | ***LYAR*** | ***DHX58*** | ***PLA2G4A*** |
| ***PDCD1*** | ***SERPING1*** | ***TYROBP*** | ***CSDE1*** | ***PTGDR*** | ***PTPRJ*** | ***TRIB1*** | ***UBASH3B*** | ***GPR137*** | ***TGFB1*** |
| ***FGL2*** | ***ADCY7*** | ***CASP3*** | ***IL27RA*** | ***ADORA2B*** | ***VSIG4*** | ***FCGR1A*** | ***MME*** | ***IRF8*** | ***FCGR3A*** |
| ***HLA-DMB*** | ***ADRB1*** | ***CD274*** | ***TBC1D10C*** | ***MC1R*** | ***LRFN5*** | ***CX3CR1*** | ***RUNX3*** | ***IRF1*** | ***CD22*** |
| ***NDFIP1*** | ***HFE*** | ***FCRLB*** | ***LCP1*** | ***NBN*** | ***CALCRL*** | ***SMAD7*** | ***AVPR2*** | ***MAP3K7*** | ***BTN2A2*** |
| ***PRKCZ*** | ***PELI1*** | ***HRH2*** | ***TMEM176A*** | ***MSH2*** | ***GATA3*** | ***PGLYRP2*** | ***IL2*** | ***ERCC1*** | ***GPR20*** |
| ***TMBIM6*** | ***B2M*** | ***NMI*** | ***STAP1*** | ***RPS6*** | ***APPL1*** | ***CNR1*** | ***TREM2*** | ***TNFSF18*** | ***IL23A*** |
| ***TMEM176B*** | ***INPP5D*** | ***CLCF1*** | ***HLA-B*** | ***IL18R1*** | ***BMP4*** | ***BLK*** | ***CCR6*** | ***MALT1*** | ***APOD*** |
| ***CR1L*** | ***ITGAL*** | ***GGT5*** | ***SUSD4*** | ***FCGR2A*** | ***PAWR*** | ***TGFB3*** | ***GHRH*** | ***GPR15*** | ***IL12RB1*** |
| ***MUL1*** | ***PTH1R*** | ***TSPAN6*** | ***CCL19*** | ***ADAM17*** | ***CD96*** | ***C4BPB*** | ***PVR*** | ***CNOT7*** | ***DAK*** |
| ***DRD5*** | ***CD55*** | ***CD68*** | ***COL3A1*** | ***NOD2*** | ***PIK3R1*** | ***CD59*** | ***YTHDF2*** | ***SDC4*** | ***YTHDF3*** |
| ***IGKV1-39*** | ***SCGB1A1*** | ***PLCG2*** | ***IGKV2D-40*** | ***EZR*** | ***FBN1*** | ***ADIPOQ*** | ***IGHV3-48*** | ***TAPBPL*** | ***BANK1*** |
| ***NLRC5*** | ***NR1H2*** | ***UNC13D*** | ***IFNE*** | ***IGHV3-30*** | ***OAS1*** | ***ZC3H12A*** | ***IGLV3-1*** | ***GPR18*** | ***PTGER4*** |
| ***UBASH3A*** | ***IFNB1*** | ***IGKV3-20*** | ***PHPT1*** | ***GIPR*** | ***IGLV2-23*** | ***PTH2R*** | ***CD1B*** | ***GCSAM*** | ***TYRO3*** |
| ***MC2R*** | ***PLCG1*** | ***KIAA0922*** | ***TNFSF13*** | ***PKN1*** | ***MSH6*** | ***IGHG4*** | ***FAM49B*** | ***C1QBP*** | ***LPXN*** |
| ***APCS*** | ***TTLL12*** | ***RIPK2*** | ***HLA-DOA*** | ***TMEM178A*** | ***GPHA2*** | ***CUEDC2*** | ***MAPK14*** | ***LIG4*** | ***GPR25*** |
| ***HLA-A*** | ***CD180*** | ***LAPTM5*** | ***PTPRC*** | ***DLL1*** | ***LTF*** | ***PRKAR1B*** | ***DPEP1*** | ***IGLV3-27*** | ***PRDX2*** |
| ***PRKACB*** | ***ADRB3*** | ***IGLV1-51*** | ***LGALS1*** | ***GPER1*** | ***PRKAR1A*** | ***CBFB*** | ***CTC-534A2.2*** | ***ADCY4*** | ***IGKV2-30*** |
| ***LHCGR*** | ***TSC22D3*** | ***CD247*** | ***ITM2A*** | ***PYCARD*** | ***IGHG3*** | ***TLR3*** | ***AKT1*** | ***MDK*** | ***CRTAM*** |
| ***CD84*** | ***TRIM21*** | ***IGKV5-2*** | ***SOX9*** | ***GPR150*** | ***LRRC17*** | ***SUV420H1*** | ***CNR2*** | ***SCRIB*** | ***FAM19A3*** |
| ***APBB1IP*** | ***HTR7*** | ***HLA-H*** | ***TRAF6*** | ***VAMP7*** | ***ERBB2*** | ***CD40*** | ***IL2RA*** | ***ZNF683*** | ***FER*** |
| ***KLRF2*** | ***MNDA*** | ***DLG1*** | ***ADCY5*** | ***VIPR1*** | ***BATF*** | ***IGKV1D-12*** | ***HSPD1*** | ***TNFRSF14*** | ***MAPKBP1*** |
| ***DGKZ*** | ***SUV420H2*** | ***FCER1G*** | ***RAMP3*** | ***HLA-DRA*** | ***MAD2L2*** | ***FCRL3*** | ***FCGR2B*** | ***NOV*** | ***RORC*** |
| ***IL23R*** | ***ZNF675*** | ***TNFAIP3*** | ***NRARP*** | ***CLNK*** | ***GLMN*** | ***GBP1*** | ***HMOX1*** | ***SOX11*** | ***PELO*** |
| ***GAPT*** | ***HLA-F*** | ***ST3GAL1*** | ***IGLV3-19*** | ***A2M*** | ***HTR6*** | ***IL6R*** | ***NLRP3*** | ***PTHLH*** | ***NLRX1*** |
| ***NME2*** | ***IGKV1-33*** | ***TRAF2*** | ***IFNL1*** | ***IGHV1-2*** | ***IFNA7*** | ***CD46*** | ***STAT6*** | ***IGKV2D-28*** | ***GPBAR1*** |
| ***NBL1*** | ***IGHV2-5*** | ***ITCH*** | ***C5*** | ***F2RL1*** | ***IGHV2-70*** | ***TCTA*** | ***IL12A*** | ***IGKV2-28*** | ***PTPN2*** |
| ***IFNG*** | ***IL21*** | ***GPR137B*** | ***PADI2*** | ***CCL28*** | ***DENND1B*** | ***IGHV3-23*** | ***CD28*** | ***FURIN*** | ***IFNA10*** |
| ***PGLYRP1*** | ***AHR*** | ***IGKV1-17*** | ***C17orf99*** | ***IGKV3-15*** | ***IL6*** | ***ANXA1*** | ***PSMB4*** | ***CD1E*** | ***CDK6*** |
| ***RNF26*** | ***C4BPA*** | ***BTK*** | ***IL12B*** | ***TIGIT*** | ***SKAP1*** | ***ADM2*** | ***DOCK10*** | ***RIF1*** | ***LGALS9B*** |
| ***DRD2*** | ***IGLV6-57*** | ***TRAF3IP1*** | ***SEMA4A*** | ***IFNW1*** | ***CREB1*** | ***IGHV1-46*** | ***NPY5R*** | ***LAG3*** | ***RC3H2*** |
| ***GLI3*** | ***PRKACA*** | ***IGKV1-16*** | ***GLP2R*** | ***EXOSC6*** | ***FSHB*** | ***ZC3H8*** | ***NOTCH2*** | ***NPLOC4*** | ***NCKAP1L*** |
| ***HLA-DOB*** | ***EMILIN1*** | ***CD300A*** | ***FOXP1*** | ***LY9*** | ***RIOK3*** | ***IGLC3*** | ***GPR83*** | ***IGHV3-11*** | ***IGLV1-40*** |
| ***CUL4A*** | ***SEC14L1*** | ***PVRL2*** | ***VAMP2*** | ***SPINK5*** | ***LILRB1*** | ***SH2D1A*** | ***FYN*** | ***IGHV3-33*** | ***IGHV3-13*** |
| ***CXCL12*** | ***CD47*** | ***TNFRSF21*** | ***XCL1*** | ***FOXJ1*** | ***IL7R*** | ***IGKV1-5*** | ***IAPP*** | ***FCER2*** | ***VIPR2*** |
| ***SWAP70*** | ***BST2*** | ***EXO1*** | ***NLRP10*** | ***IGHV3-7*** | ***DUSP1*** | ***SHH*** | ***KLRD1*** | ***TP53BP1*** | ***TNFSF13B*** |
| ***DDT*** | ***CST7*** | ***IL31RA*** | ***CD300LF*** | ***GCG*** | ***PAXIP1*** | ***PRKAR2A*** | ***ADM*** | ***ARG1*** | ***PPM1B*** |
| ***IL4R*** | ***VTCN1*** | ***FOXF1*** | ***LILRB4*** | ***CAD*** | ***PLA2G2D*** | ***AXL*** | ***SLAMF8*** | ***GPNMB*** | ***CLEC12B*** |
| ***ICOS*** | ***RORA*** | ***ADORA1*** | ***ZBTB46*** | ***CR1*** | ***LAMP1*** | ***PLK2*** | ***MYC*** | ***THBS1*** | ***IGHG1*** |
| ***FADD*** | ***RLN2*** | ***SOCS5*** | ***C10orf54*** | ***IGHG2*** | ***CRHR2*** | ***HPX*** | ***GAL*** | ***VIMP*** | ***SAMSN1*** |
| ***SLC22A13*** | ***PRKACG*** | ***HOXA7*** | ***CLEC6A*** | ***CCR2*** | ***GREM1*** | ***SIRT2*** | ***EXOSC3*** | ***TGFB2*** | ***MYH9*** |
| ***ADCY2*** | ***ADA*** | ***ADCYAP1*** | ***PLA2G2F*** | ***SLC15A4*** | ***DCST1*** | ***TARM1*** | ***XBP1*** | ***ADCY9*** | ***SLC11A1*** |
| ***NR1H3*** | ***PLCB1*** | ***TSC1*** | ***CD160*** | ***KIR2DL4*** | ***CD81*** | ***SERPINB4*** | ***IGHV4-59*** | ***CR2*** | ***IGHV3-53*** |
| ***ZPBP2*** | ***IGLV2-14*** | ***ARRB2*** | ***RXFP1*** | ***STX7*** | ***CCL3*** | ***ATM*** | ***APOA2*** | ***SLAMF1*** | ***CRK*** |
| ***IGLC6*** | ***GHRHR*** | ***RNF8*** | ***IGHV4-34*** | ***AP1G1*** | ***ADCY3*** | ***PTGER2*** | ***IL20RB*** | ***ADTRP*** | ***PTGIR*** |
| ***MC5R*** | ***INHBA*** | ***VIP*** | ***IGKV1-12*** | ***CLDN18*** | ***LILRB3*** | ***C5AR2*** | ***LGALS3*** | ***SUPT6H*** | ***FSTL3*** |
| ***PTPN22*** | ***PARP3*** | ***FBXW7*** | ***KLRC1*** | ***ISG15*** | ***IGLV7-43*** | ***CX3CL1*** | ***HLA-E*** | ***TREX1*** | ***PRKDC*** |
| ***GPX1*** | ***DUSP10*** | ***AMBP*** | ***IGKV1D-39*** | ***RPS19*** | ***PARP14*** | ***SPI1*** | ***USP15*** | ***PSEN1*** | ***CD200*** |
| ***CARD9*** | ***IGKV4-1*** | ***CD74*** | ***HLX*** | ***LTA*** | ***ID2*** | ***CEACAM1*** | ***TNFSF4*** | ***CNOT6*** | ***IGKV3D-20*** |
| ***SLA2*** | ***WHSC1*** | ***RC3H1*** | ***GATA2*** | ***CALCA*** | ***MICA*** | ***INSL3*** | ***TP53*** | ***CTNNB1*** | ***IGLV1-44*** |
| ***CCL2*** | ***LST1*** | ***AZGP1*** | ***IGHV4-39*** | ***PLCL2*** | ***IL17D*** | ***STAT3*** | ***MYB*** | ***HLA-DPB1*** | ***MAD1L1*** |
| ***IGKC*** | ***ATP7A*** | ***TFRC*** | ***CD80*** | ***FBXO38*** | ***IGKV1D-16*** | ***RAMP2*** | ***IGKV3-11*** | ***IL4I1*** | ***PIAS3*** |
| ***GPR55*** | ***IGLC2*** | ***IGLC1*** | ***SFRP1*** | ***ENTPD7*** | ***ADCY6*** | ***C20orf196*** | ***MICB*** | ***FAM35A*** | ***PPARG*** |
| ***IGLV3-21*** | ***CD244*** | ***APOA1*** | ***MTOR*** | ***METTL3*** | ***LYN*** | ***MR1*** | ***STAT2*** | ***KLRC2*** | ***DRD4*** |
| ***DRD3*** | ***USP18*** | ***HLA-DRB1*** | ***CD4*** | ***IGLV3-25*** | ***GLP1R*** | ***SLAMF6*** | ***BCL6*** | ***HCK*** | ***IGKV2-29*** |
| ***LGALS9*** | ***HTR4*** | ***IGLV1-47*** | ***PVRIG*** | ***CALCB*** | ***GPR176*** | ***MMP28*** | ***ZP3*** | ***IGHV3-9*** | ***ARG2*** |
| ***RLN3*** | ***UNG*** | ***IGHV1-69*** | ***LRCH1*** | ***GGT1*** | ***IL27*** | ***GPR39*** | ***IFNK*** | ***GPR68*** | ***CEBPB*** |
| ***ICAM1*** | ***GPR45*** | ***GRN*** | ***IGLV2-8*** | ***DRD1*** | ***CTLA4*** | ***TARBP2*** | ***CCL21*** | ***SOCS1*** | ***ADAR*** |
| ***SPN*** | ***TNFAIP8L2*** | ***IGKV2D-30*** | ***BPI*** | ***FGR*** | ***MLH1*** | ***HSPA9*** | ***MIA3*** | ***ABL1*** | ***LRRC32*** |
| ***IHH*** | ***YES1*** | ***NPSR1*** | ***LDLR*** | ***UFD1L*** | ***GPR183*** | ***EIF2AK4*** | ***ADCYAP1R1*** | ***LHB*** | ***HLA-G*** |
| ***RXFP2*** | ***CALCR*** | ***GPS2*** | ***LILRB2*** | ***PCK1*** | ***NLRP6*** | ***MIF*** | ***LAX1*** | ***CRHR1*** | ***TSHR*** |
| ***RNF168*** | ***SIRT1*** | ***SCT*** | ***MILR1*** | ***BMP5*** | ***FSHR*** | ***C6orf106*** | ***POMC*** | ***GPR84*** | ***IGLV2-11*** |
| ***ENPP3*** | ***GPR32*** | ***RHBDD3*** | ***IGLC7*** | ***CYSLTR1*** | ***GPAM*** | ***LGALS7*** | ***CARTPT*** | ***GIP*** | ***PTH2*** |
| ***NPS*** | ***ADCY8*** | ***INS*** | ***PTH*** | ***TSHB*** | ***IGKV1D-33*** | ***CGA*** | ***AVP*** | ***MC3R*** | ***IFNA2*** |
| ***FGL1*** | ***TAAR1*** | ***IFNA8*** | ***IFNA16*** | ***IFNA4*** | ***IFNA6*** | ***IFNA14*** | ***IFNA1*** | ***NKX2-3*** | ***IFNA21*** |
| ***IFNA5*** | ***IFNA17*** |  |  |  |  |  |  |  |  |
